# Artificial Hunyadi Janos Water

**Published:** 1882-03

**Authors:** 


### ARTIFICIAL HUNYADI JANOS WATER.

The natural Hunyadi Janos water was observed to be an efficient, safe, and agreeable purgative in many chronic cases. It is, however, found to be too expensive for hospital use, and it was resolved to try it artificially. At first it was made according to Liebig's analysis of the natural water, but this was perceived to be to weak, and it failed to produce purgative action. Ultimately it was made thrice the given strength according to the following recipe: Sulphate of magnesia, 514.92 gr.; sulphate of soda, 519.54 gr.; sulphate of potash, 2.76 gr.; chloride of sodium, 39.15 gr.; bicarbonate of soda, 15.60 gr.; water, 16 oz. Dose, two ounces and upward. It will be observed that the chloride of calcium is omitted, but the proportion is so small that even when it was included there was no difference in the action. This inexpensive mixture, made for a penny per quart, can be effectually recommended. It will be found to possess every advantage attributed to the natural variety, the necessity for buying which seems to be done away with.—*London Lancet.*

